# Tumor-specific *KCNJ5* mutation in an aldosterone-producing adenoma not detected in adrenal venous plasma cell-free DNA

**DOI:** 10.1210/jcemcr/luag222

**Published:** 2026-08-28

**Authors:** Daisuke Aono, Toshiaki Kato, Mitsuhiro Kometani, Takashi Yoneda, Kazuyoshi Hosomichi, Shigehiro Karashima

**Affiliations:** Department of Health Promotion and Medicine of the Future, Graduate School of Medical Sciences, Kanazawa University, Kanazawa, Ishikawa 920-8641, Japan; Department of Health Promotion and Medicine of the Future, Graduate School of Medical Sciences, Kanazawa University, Kanazawa, Ishikawa 920-8641, Japan; Department of Health Promotion and Medicine of the Future, Graduate School of Medical Sciences, Kanazawa University, Kanazawa, Ishikawa 920-8641, Japan; Department of Health Promotion and Medicine of the Future, Graduate School of Medical Sciences, Kanazawa University, Kanazawa, Ishikawa 920-8641, Japan; Laboratory of Computational Genomics, School of Life Science, Tokyo University of Pharmacy and Life Sciences, Tokyo 192-0392, Japan; Institute of Liberal Arts and Science, Kanazawa University, Kanazawa, Ishikawa 920-1192, Japan

**Keywords:** aldosterone-producing adenoma, primary aldosteronism, circulating tumor DNA, *KCNJ5* somatic mutation, adrenal venous sampling

## Abstract

Circulating tumor DNA (ctDNA) can be detected in adrenocortical carcinoma; however, its detectability in benign adrenal tumors, such as aldosterone-producing adenoma (APA), remains unclear. We investigated whether tumor-specific mutations could be detected in plasma cell-free DNA (cfDNA) obtained from peripheral and adrenal venous blood in a 34-year-old man with APA. The patient was evaluated for secondary hypertension, and screening and confirmatory tests established the diagnosis of primary aldosteronism. Computed tomography revealed an 11-mm left adrenal mass, and adrenal venous sampling demonstrated left-sided aldosterone hypersecretion. The patient subsequently underwent left adrenalectomy, and pathological examination confirmed an APA. Targeted next-generation sequencing of 18 adrenal disease–related genes identified a somatic *KCNJ5* mutation in the tumor tissue. However, the corresponding mutation was not detectable in cfDNA from either peripheral or adrenal venous plasma, despite sampling under clinically favorable conditions. Postoperatively, blood pressure and serum potassium levels normalized. This case illustrates the challenges of detecting tumor-derived mutations in plasma from small benign adrenal tumors using standard sequencing approaches and highlights how tumor burden, sampling conditions, and assay sensitivity influence ctDNA detectability. Further studies employing highly sensitive, mutation-targeted methods are warranted to elucidate the biological and technical factors affecting ctDNA detection in APA.

## Introduction

Primary aldosteronism (PA) is a major cause of secondary hypertension and is associated with increased risk of cardiovascular morbidity, including atrial fibrillation, left ventricular hypertrophy, and stroke [1]. Early diagnosis and accurate subtype classification are crucial for preventing long-term complications. Among the subtypes of PA, aldosterone-producing adenoma (APA) is clinically important because it is surgically curable, whereas bilateral adrenal hyperplasia requires medical therapy. Thus, accurate identification of APA is essential for appropriate management.

Recent molecular studies have identified recurrent somatic mutations in APAs, most notably in the *KCNJ5* gene, that contribute to autonomous aldosterone production [2, 3]. However, these analyses require resected tumor tissue, and noninvasive approaches for detecting tumor-specific mutations before surgery remain limited.

There is a biological rationale for exploring tumor-derived DNA in blood, as circulating cell-free DNA (cfDNA) in plasma may contain tumor-specific mutations depending on tumor biology and assay sensitivity [4, 5].

In APA, the small tumor burden and benign nature may limit circulating tumor DNA (ctDNA) release into the circulation, potentially reducing its detectability in plasma. Previous studies have primarily focused on peripheral blood, where ctDNA detection remains challenging [6]. In this context, adrenal venous blood may represent a theoretically enriched source of ctDNA, as it is obtained directly from the adrenal gland.

Although adrenal venous sampling (AVS) is a cornerstone for identifying unilateral aldosterone excess, ctDNA analysis is not intended to replace AVS; rather, when detectable, it may provide complementary molecular information in selected settings. Whether such information can be reliably obtained in APA using current methodologies remains unclear.

We previously demonstrated the utility of ctDNA analysis in adrenocortical carcinoma [7]; however, data on benign adrenal tumors, including APA, remain limited. Therefore, we aimed to investigate whether tumor-specific mutations could be detected in plasma cfDNA from peripheral and adrenal venous blood in a patient with APA using a targeted next-generation sequencing (NGS) panel including *KCNJ5*. In this report, we analyzed plasma cfDNA to assess the detectability of tumor-specific mutations in ctDNA.

## Case presentation

A 34-year-old man had a history of hypertension first noted in his late twenties, managed with lifestyle modification alone. Home blood pressure was in the 140s/80s mmHg. One year before referral, hypertension and hypokalemia were identified during a routine medical examination, prompting further evaluation. There was no relevant family history of endocrine disorders.

## Diagnostic assessment

**Figure 1:**
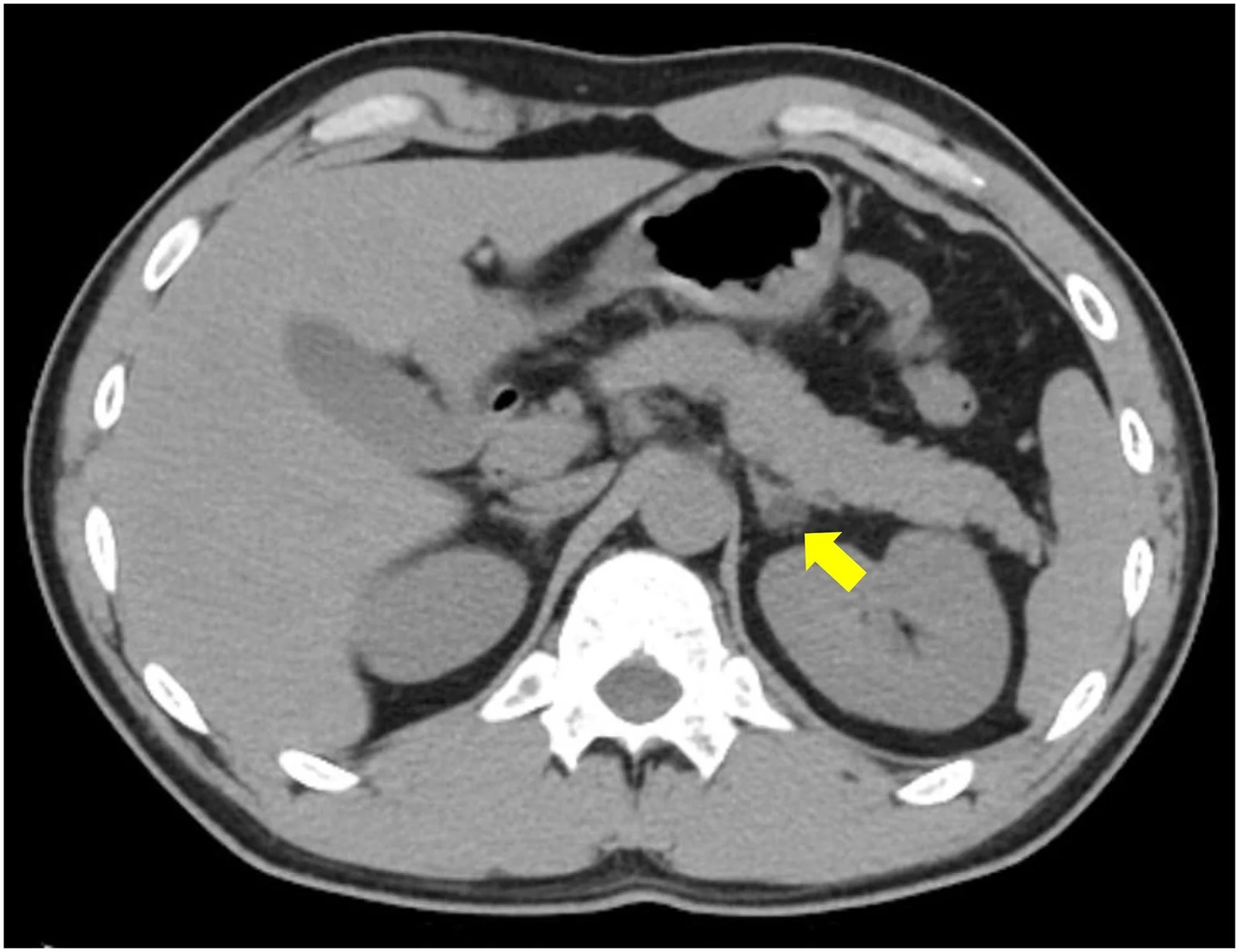
Abdominal CT scan. An unenhanced abdominal CT scan showing a 1.1-cm left adrenal tumor (arrow) with an attenuation value of −10 Hounsfield units.

At presentation, blood pressure was 119/65 mmHg while receiving a dihydropyridine calcium channel blocker (cilnidipine, 10 mg twice daily), and serum potassium was maintained at the lower limit of normal (3.5 mEq/L [SI: 3.5 mmol/L]; reference range, 3.5-5.0 mEq/L [SI: 3.5-5.0 mmol/L]) using oral potassium chloride slow-release tablets (600 mg once daily) (Table 1). Physical examination was unremarkable. Endocrinological evaluation showed a plasma renin activity of 0.7 ng/mL/h (reference range, 0.2-2.7 ng/mL/h) and a plasma aldosterone concentration of 20.2 ng/dL (SI: 560 pmol/L; reference range, 4.0-31.0 ng/dL [SI: 111-861 pmol/L]), yielding an aldosterone-to-renin ratio of 28.8, consistent with PA (Table 2). Confirmatory testing, including the captopril challenge, saline infusion, and upright furosemide tests, was positive. A 1-mg overnight dexamethasone suppression test showed adequate cortisol suppression (0.37 µg/dL [SI: 10.2 nmol/L]; reference range, <1.8 µg/dL [SI: <50 nmol/L]), arguing against autonomous cortisol secretion. Abdominal computed tomography revealed an 11-mm left adrenal mass with an unenhanced attenuation value of −10 Hounsfield units (Fig. 1). AVS demonstrated successful catheterization with appropriate selectivity indices and left-sided lateralization before and after adrenocorticotropic hormone stimulation, confirming adequate and reliable AVS (Table 3).

**Table 1:** Laboratory data

Parameters	Values	Reference range
**Blood routine**		
White blood cells	4.87 × 10^9^/L (4.87 × 10^3^/µL)	3.30-8.60 × 10^9^/L (3.30-8.60 × 10^3^/µL)
Red blood cells	5.25 × 10^12^/L (5.25 × 10^6^/µL)	4.35-5.55 × 10^12^/L (4.35-5.55 × 10^6^/µL)
Hemoglobin	152 g/L (15.2 g/dL)	135-176 g/L (13.5-17.6 g/dL)
Platelets	263 × 10^9^/L (263 × 10^4^/µL)	158-348 × 10^9^/L (158-348 × 10^4^/µL)
**Biochemistry**		
Total protein	67 g/L (6.7 g/dL)	67-83 g/L (6.7-8.3 g/dL)
Albumin	42 g/L (4.2 g/dL)	40-50 g/L (4.0-5.0 g/dL)
Aspartate aminotransferase	20 U/L	13-33 U/L
Alanine aminotransferase	24 U/L	8-42 U/L
Blood urea nitrogen	3.6 mmol/L (10 mg/dL)	2.9-7.9 mmol/L (8.0-22.0 mg/dL)
Creatinine	61 µmol/L (0.69 mg/dL)	53-88 µmol/L (0.60-1.00 mg/dL)
Sodium	146 mmol/L (146 mEq/L)	135-149 mmol/L (135-149 mEq/L)
Potassium	3.5 mmol/L (3.5 mEq/L)	3.5-4.9 mmol/L (3.5-4.9 mEq/L)
Chloride	106 mmol/L (106 mEq/L)	96-108 mmol/L (96-108 mEq/L)
Calcium	2.25 mmol/L (9.0 mg/dL)	2.0-2.6 mmol/L (8.0-10.5 mg/dL)
Phosphate	0.68 mmol/L (2.1 mg/dL)	0.81-1.45 mmol/L (2.5-4.5 mg/dL)
Total cholesterol	5.93 mmol/L (229 mg/dL)	3.31-5.67 mmol/L (128-219 mg/dL)
Triglycerides	2.44 mmol/L (216 mg/dL)	0.34-1.68 mmol/L (30-149 mg/dL)
HDL cholesterol	1.40 mmol/L (54 mg/dL)	1.03-2.56 mmol/L (40-99 mg/dL)
**Glucose metabolism**		
Fasting blood glucose	5.44 mmol/L (98 mg/dL)	3.83-6.05 mmol/L (69-109 mg/dL)
Hemoglobin A1c	5.1%	4.6-6.2%
**Urinary excretion**		
Protein (qualitative)	Negative	Negative
Glucose (qualitative)	Negative	Negative
Blood (qualitative)	Negative	Negative

Values are presented in SI units with corresponding conventional units in parentheses.

Abbreviation: HDL, high-density lipoprotein.

**Table 2:** Endocrinological examinations

Parameters	Values	Reference range	
**Fasting blood test**			
TSH	0.66 mIU/L (0.66 µU/mL)	0.27-4.20 mIU/L (0.27-4.20 µU/mL)	
Free T3	5.34 pmol/L (3.47 pg/mL)	3.54-6.16 pmol/L (2.30-4.00 pg/mL)	
Free T4	19.0 pmol/L (1.47 ng/dL)	12.9-23.2 pmol/L (1.00-1.80 ng/dL)	
Parathyroid hormone	7.2 pmol/L (67.9 pg/mL)	1.1-7.0 pmol/L (10.3-65.9 pg/mL)	
Adrenocorticotropic hormone	13.4 pmol/L (60.9 pg/mL)	≤10.1 pmol/L (≤46 pg/mL)	
Cortisol	472 nmol/L (17.1 µg/dL)	171-535 nmol/L (6.2-19.4 µg/dL)	
DHEA-S	4.08 µmol/L (151 µg/dL)	2.87-12.5 µmol/L (106-464 µg/dL)	
Metanephrine	354 pmol/L (69.9 pg/mL)	≤659 pmol/L (≤130.0 pg/mL)	
Normetanephrine	958 pmol/L (175.5 pg/mL)	≤2763 pmol/L (≤506.0 pg/mL)	
Plasma aldosterone concentration	560 pmol/L (20.2 ng/dL)	111-861 pmol/L (4.0-31.0 ng/dL)	
Plasma renin activity	0.7 ng/mL/h	0.2-2.7 ng/mL/h	
**24-hour urine collection test**			
Metanephrine	812 nmol/day (0.16 mg/day)	254-1015 nmol/day (0.05-0.20 mg/day)	
Normetanephrine	1146 nmol/day (0.21 mg/day)	546-1528 nmol/day (0.10-0.28 mg/day)	
**Captopril loading test**			
**Parameters**	**0 minutes**	**60 minutes**	**90 minutes**
Plasma aldosterone concentration	266 pmol/L (9.6 ng/dL)	244 pmol/L (8.8 ng/dL)	252 pmol/L (9.1 ng/dL)
Plasma renin activity	0.3 ng/mL/h	0.4 ng/mL/h	0.6 ng/mL/h
Aldosterone-to-renin ratio	321	220	151
**Saline infusion test**			
**Parameters**	**0 minutes**	**120 minutes**	
Plasma aldosterone concentration	313 pmol/L (11.3 ng/dL)	291 pmol/L (10.5 ng/dL)	
**Upright furosemide test**			
**Parameters**	**0 minutes**	**90 minutes**	
Plasma renin activity	0.3 ng/mL/h	0.9 ng/mL/h	
**1-mg overnight dexamethasone suppression test**			
**Parameters**	**Values**	**Reference range**	
Cortisol	10.2 nmol/L (0.37 µg/dL)	<50 nmol/L (<1.8 µg/dL)	

Values are presented in SI units with corresponding conventional units in parentheses.

Interpretation was based on standard diagnostic criteria for primary aldosteronism.

Abbreviations: DHEA-S, dehydroepiandrosterone sulfate; Free T3, free triiodothyronine; Free T4, free thyroxine; TSH, thyroid-stimulating hormone.

**Table 3:** Results of adrenal venous sampling before and after ACTH stimulation

Sample	Aldosterone	Cortisol	SI	LI
**Before ACTH stimulation**				
IVC	224 pmol/L (8.1 ng/dL)	312 nmol/L (11.3 µg/dL)	—	—
RAV	399 pmol/L (14.4 ng/dL)	1192 nmol/L (43.2 µg/dL)	3.8	—
LAV	5756 pmol/L (207.9 ng/dL)	676 nmol/L (24.5 µg/dL)	2.2	25.5
**After ACTH stimulation**				
IVC	491 pmol/L (17.7 ng/dL)	466 nmol/L (16.9 µg/dL)	—	—
RAV	3281 pmol/L (118.8 ng/dL)	23 819 nmol/L (863.0 µg/dL)	51.1	—
LAV	47 545 pmol/L (1720.8 ng/dL)	20 755 nmol/L (752.0 µg/dL)	44.5	16.6

Blood samples were collected 30 minutes after a bolus intravenous injection of ACTH (tetracosactide, 0.25 mg = 25 IU) during ACTH-loading adrenal venous sampling.

Values are presented in SI units with corresponding conventional units in parentheses.

Abbreviations: ACTH, adrenocorticotropic hormone; IVC, inferior vena cava; LAV, left adrenal vein; LI, lateralization index = (aldosterone/cortisol ratio of dominant adrenal vein)/(aldosterone/cortisol ratio of contralateral adrenal vein); RAV, right adrenal vein; SI, selectivity index = cortisol of adrenal vein/cortisol of inferior vena cava.

## Treatment

**Figure 2:**
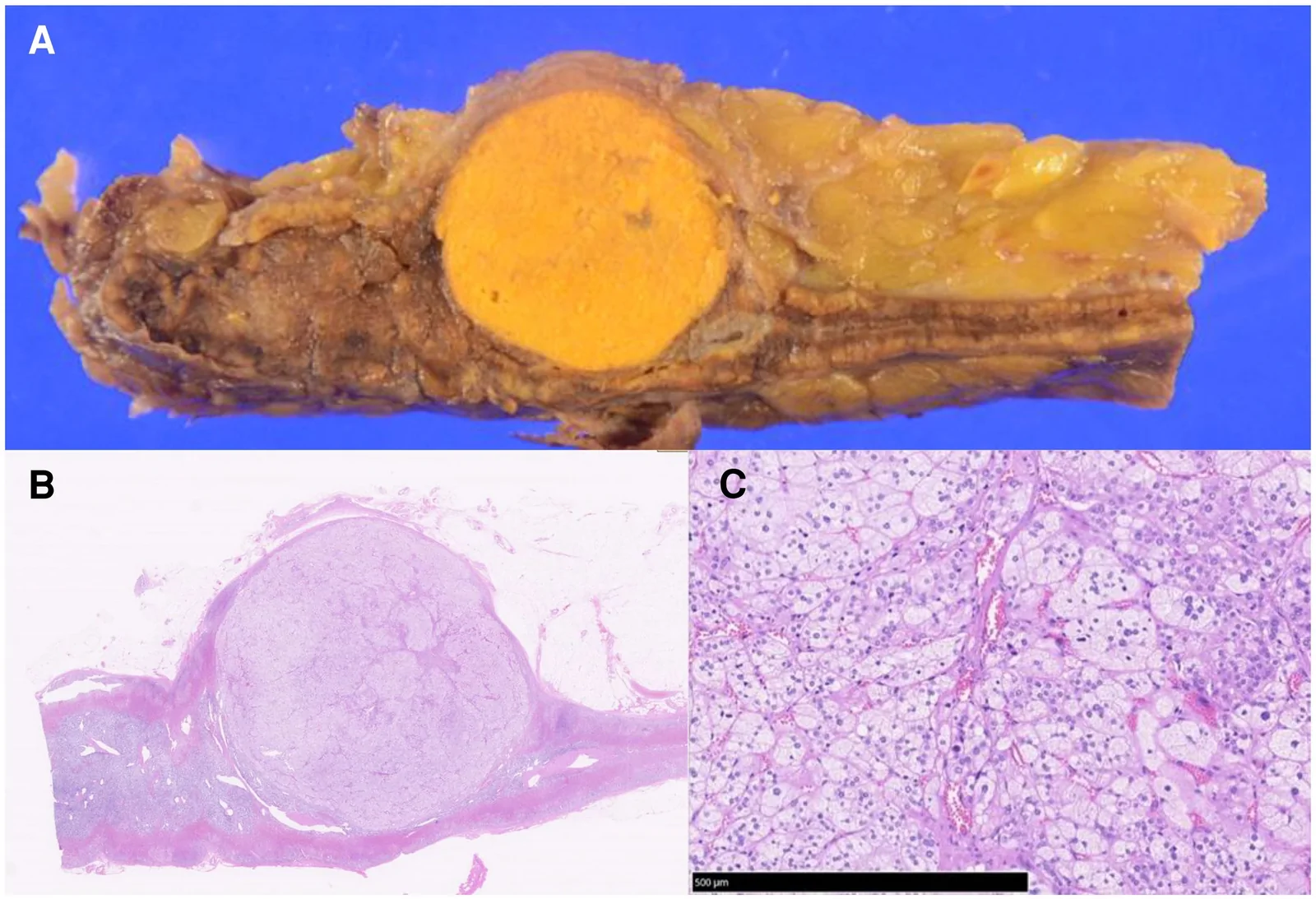
Histopathological examination. (A) Macroscopic view of the resected left adrenal tumor measuring 1.2 cm. (B, C) Hematoxylin–eosin staining showing features consistent with adrenocortical adenoma (B, ×1; C, ×10).

Based on AVS findings, the patient underwent left adrenalectomy. Macroscopic and histopathological images of the resected 1.2-cm left adrenal tumor. The gross specimen shows a well-circumscribed, solid, yellow nodule, and hematoxylin–eosin stained sections at ×1 and ×10 magnification show features consistent with an adrenocortical adenoma (Fig. 2). Histopathological evaluation confirmed an adrenocortical adenoma compatible with an APA. Immunohistochemistry was not performed.

To assess whether tumor-derived mutations were detectable in circulation, plasma cfDNA from peripheral and adrenal venous blood obtained during AVS was analyzed, along with tumor DNA obtained after adrenalectomy. cfDNA was isolated from 2 mL of plasma per sample, obtained from preoperative peripheral blood and adrenal venous blood collected during AVS, using Streck Cell-Free DNA BCT tubes. Targeted NGS was performed using an adrenal disease–related 18-gene panel (*APC*, *ARMC5*, *ATP1A1*, *ATP2B3*, *CACNA1D*, *CACNA1H*, *CTNNB1*, *CYP11B1*, *GNAS*, *HSD3B2*, *KCNJ5*, *MEN1*, *NF1*, *PDE1A*, *PRKACA*, *PRKAR1A*, *RET*, and *VHL*) on DNA extracted from the resected tumor and adjacent normal adrenal tissue, as well as on cfDNA. Library preparation, sequencing, and variant calling were performed as previously described [7].

Sequencing identified a somatic *KCNJ5* mutation (c.451G>A; p.G151R) in the tumor (variant allele fraction, 12%) (Table 4). The mutation was absent in adjacent normal tissue and was not detected in cfDNA from either peripheral or adrenal venous plasma. In adrenal venous plasma obtained under technically adequate AVS conditions, sufficient sequencing depth was achieved at the *KCNJ5* locus; however, the tumor-specific mutation was not detected.

**Table 4:** Genetic mutation analysis in APA tissue and matched blood samples

Sample			DNA concentration	Genetic mutations	DNA quality	Allelic number	NGS depth	NGS allelic fraction
Tissue	Adrenal tumor		129 ng/mL	*KCNJ5* c.451G>A; p.G151R	NA	38	315	12%
	Adjacent normal adrenal gland		167 ng/mL	—	NA	NA	311	NA
	Surrounding adipose tissue		37.6 ng/mL	—	NA	NA	323	NA
Blood	Peripheral blood		74.5 ng/mL	—	91%	NA	354	NA
		IVC	71.4 ng/mL	—	86%	NA	745	NA
	AVS	RAV	85.7 ng/mL	—	90%	NA	425	NA
	before ACTH stimulation	LAV	126 ng/mL	—	93%	NA	431	NA
		IVC	116 ng/mL	—	87%	NA	409	NA
		IVC	113 ng/mL	—	85%	NA	360	NA
	AVS	RAV	106 ng/mL	—	93%	NA	459	NA
	after ACTH stimulation	LAV	101 ng/mL	—	96%	NA	311	NA
		IVC	113 ng/mL	—	84%	NA	364	NA

Abbreviations: —, no mutation detected; ACTH, adrenocorticotropic hormone; AVS, adrenal venous sampling; IVC, inferior vena cava; LAV, left adrenal vein; NA, not available; NGS, next-generation sequencing; RAV, right adrenal vein.

## Outcome and follow-up

At 12 months postoperatively, the patient met the Primary Aldosteronism Surgical Outcome criteria for complete clinical success, with normalization of blood pressure (115/69 mmHg) without antihypertensive medication [8]. Biochemical evaluation demonstrated normalization of serum potassium (4.0 mEq/L [SI: 4.0 mmol/L]) without potassium supplementation and an aldosterone–renin profile consistent with complete biochemical success (plasma aldosterone concentration: 13.2 ng/dL [SI: 37 pmol/L]; plasma renin activity: 0.6 ng/mL/h), with no evidence of recurrent hyperaldosteronism. This favorable outcome was maintained for 3 years of follow-up.

## Discussion

We assessed whether a tumor-specific mutation could be detected as ctDNA in plasma cfDNA from a patient with APA by analyzing peripheral and adrenal venous plasma. This case was not selected based on age or expected mutation status but represents the only case in our cohort in which technically successful AVS and subsequent adrenalectomy were performed, allowing comprehensive evaluation of tumor tissue and paired plasma samples. Targeted sequencing identified a somatic *KCNJ5* mutation in the resected tumor; however, the same mutation was not detected in cfDNA from either peripheral or adrenal venous plasma.

Adrenal venous blood was obtained under reliable conditions, with successful catheterization and appropriate selectivity indices before and after adrenocorticotropic hormone stimulation. AVS represents a theoretical best-case scenario for ctDNA detection in APA, as blood is collected directly from the adrenal vein. Therefore, the absence of detectable tumor-specific mutations under these conditions strongly suggests that the negative result reflects fundamental biological and analytical limitations—such as low tumor DNA shedding and insufficient assay sensitivity—rather than suboptimal sampling or technical failure. These findings can also be interpreted from a pathophysiological perspective: AVS reflects lateralized hormone secretion, whereas ctDNA analysis captures the tumor clone harboring the driver mutation, highlighting that hormone secretion and tumor DNA shedding are not necessarily proportional.

Consistent with our findings, a prior study of APA (n = 5; Cases #2-6 in Table 5) reported that although plasma cfDNA was measurable, tumor-specific mutations were not detected in peripheral blood in any patient [6]. Notably, tumor mutations were present in 3 of these cases, yet remained undetectable in plasma, highlighting the analytical challenge of detecting very low–fraction variants in benign adrenal disease using standard-coverage targeted NGS. In contrast, the present study evaluated adrenal venous samples—representing a near-ideal condition for ctDNA enrichment—and similarly failed to detect tumor-specific mutations. This comparison suggests that the limitation of ctDNA detection is not solely attributable to sampling site but reflects intrinsic biological and analytical constraints.

**Table 5:** ctDNA analysis in patients with APA

Case # [Ref]	Age/sex	CT findings	AVS laterality	Genetic mutations		
				Adrenal venous blood	Peripheral blood	Tumor tissue
1 [present]	34/M	11 mm left adrenal mass	Left-sided dominance	ND	ND	*KCNJ5* c.451G>A; p.G151R (pathogenic/likely pathogenic)
2 [6]	NA	NA	NA	NA	ND	ND
3 [6]	NA	NA	NA	NA	ND	ND
4 [6]	NA	NA	NA	NA	ND	*KCNJ5* c.451G>A; p.G151R (pathogenic/likely pathogenic)
5 [6]	NA	NA	NA	NA	ND	*KCNJ5* c.503T>G; p.L168R (likely pathogenic)
6 [6]	NA	NA	NA	NA	ND	*CACNA1D* c.3044T>C; I1015T (unknown)

Abbreviations: APA, aldosterone-producing adenoma; AVS, adrenal venous sampling; CT, computed tomography; ctDNA, circulating tumor DNA; F, female; M, male; NA, not available; ND, not detected.

A broad adrenal disease–related gene panel was used to comprehensively assess adrenal tumors; however, in clinically typical APA cases, a more focused approach may be sufficient. Somatic mutations in APAs include several recurrent genes, among which *KCNJ5* is the most frequently reported [2, 3, 9]. APAs harboring *KCNJ5* mutations have been associated with more favorable postoperative outcomes after adrenalectomy [9]. Therefore, if tumor-specific mutations such as *KCNJ5* could be reliably identified preoperatively, they may provide clinically useful information. Although AVS is the standard method for subtype classification in PA, its interpretation can be challenging in a subset of patients, occasionally resulting in equivocal findings. In such cases, ctDNA analysis may provide complementary molecular information reflecting the biological basis of aldosterone excess. However, our findings suggest that despite this potential clinical value, implementation of ctDNA analysis using standard-coverage targeted NGS remains challenging. In addition, although ctDNA analysis may be useful in selected clinical scenarios, its routine application in cases with clear lateralization may be limited, particularly when considering cost-effectiveness.

More broadly, ctDNA detectability in benign tumors and premalignant lesions remains debated. Benign tumors release less ctDNA than malignant tumors [10]. Studies of colorectal adenomas and polyps show that tumor-associated mutations can be detected in plasma ctDNA, although sensitivity is low [11, 12]. Similarly, in pituitary adenomas, such mutations are often undetectable in plasma because of limited analytical sensitivity [13]. Collectively, these data support that benign or low-grade lesions may shed ctDNA, typically below the detection threshold of standard-coverage targeted NGS. ctDNA detectability in benign tumors may depend on factors such as tumor burden and accompanying tissue injury. Given that the tumor in the present case was of average size for APA (approximately 12 mm) and showed no overt features, such as necrosis or hemorrhage, that would facilitate tumor DNA release, the absence of detectable ctDNA is not unexpected.

This report has some limitations. First, the present report includes only a single APA case with tumor tissue confirmation, limiting generalizability. Second, the negative circulating result is constrained by assay sensitivity and preanalytical factors, including plasma input volume, cfDNA yield, library complexity, and dilutional effects. Even adrenal venous samples may be affected by sampling conditions and admixture, and the variant allele fraction in the tumor (12%) may translate into an extremely low fractional abundance in plasma. Although a similar NGS-based approach detected ctDNA in adrenocortical carcinoma [7], it may be insufficient for benign adrenal lesions. Future work could incorporate highly sensitive, mutation-targeted approaches (eg, focused sequencing or digital PCR targeting *KCNJ5* hotspots) with explicit reporting of analytical limits of detection and quality control metrics to better define when, if ever, ctDNA becomes detectable in APA.

In conclusion, this case does not demonstrate the absence of ctDNA in APA. Rather, it delineates a practical upper bound for ctDNA detection under clinically favorable and commonly used conditions, namely, standard targeted NGS, a plasma input volume of 2 mL, and sampling from the adrenal vein during technically successful AVS. These findings may inform future study design by clarifying the analytical sensitivity, plasma volume, and methodological refinements required to improve ctDNA detection in benign adrenal disease. Furthermore, this case can be considered an exploratory evaluation of ctDNA detectability during AVS under clinically optimal conditions.

## Learning points

- Tumor-specific mutations identified in APA tissue may not be detectable in plasma cfDNA using standard targeted NGS, even under favorable conditions, including adrenal venous sampling.
- Negative ctDNA results do not exclude the presence of tumor-derived DNA in APA; rather, they should be interpreted in the context of assay sensitivity, plasma input volume, and tumor burden.

## Data Availability

Some or all datasets generated during and/or analyzed during the current study are not publicly available but are available from the corresponding author on reasonable request.

## Abbreviations

APA: aldosterone-producing adenoma
AVS: adrenal venous sampling
cfDNA: cell-free DNA
ctDNA: circulating tumor DNA
NGS: next-generation sequencing
PA: primary aldosteronism
